# Antenatal counseling on breastfeeding – is it adequate? A descriptive study from Pondicherry, India

**DOI:** 10.1186/1746-4358-3-5

**Published:** 2008-03-04

**Authors:** Gunasekaran Dhandapany, Adhisivam Bethou, Arulkumaran Arunagirinathan, Shanthi Ananthakrishnan

**Affiliations:** 1 Department of Pediatrics, Mahatma Gandhi Medical College and Research Institute, Pondicherry, India

## Abstract

**Background:**

Antenatal counseling on breastfeeding and postnatal lactation support are likely to improve rates of exclusive breastfeeding. This descriptive study was undertaken to assess whether antenatal visits were utilized for promotion of exclusive breastfeeding in addition to the routine obstetric services.

**Methods:**

This descriptive study was conducted at a tertiary hospital in Pondicherry, India. Every third primigravida mother admitted in the maternity ward from June to December 2005 was recruited. Among these 144 primigravida mothers, 108 who had a minimum of three antenatal visits ("booked") were included in the study. These 108 mothers were administered a pre-tested semi-structured questionnaire on breastfeeding in the local language, Tamil, within 24 hours of giving birth. Appropriate flash cards with pictures were also used while administering the questionnaire. The awareness among mothers (both "counseled" and "not counseled") regarding health information pertaining to breastfeeding was assessed.

**Results:**

Of the booked mothers, 21% (n = 23) had received some antenatal counseling about breastfeeding while 79% (n = 85) had not received any such counseling. Four percent had undergone breast examination during antenatal visits. Awareness related to breastfeeding among mothers in the "counseled" group was better than those in the "not counseled" group. Even in the "counseled" group, awareness among mothers with regard to correct breastfeeding technique and concept of continuing breastfeeding during illness in the baby was no different from those in the "not counseled" group.

**Conclusion:**

Existing antenatal counseling on breastfeeding is inadequate in the population studied and needs to be strengthened. Informing all pregnant women about the benefits and management of breastfeeding should be a priority during antenatal visits.

## Background

The promotion and support of breastfeeding is a global priority and an important child-survival intervention and the World Health Organization advocates exclusive breastfeeding for six months. However, in reality many mothers are unable to practice exclusive breastfeeding as advocated. Lack of confidence in mothers' ability to breastfeed, problems with the infant latching or suckling, breast pain or soreness, perceptions of insufficient milk supply, and a lack of individualized encouragement from their clinicians in the early post discharge period are some of the common reasons for early breastfeeding discontinuation. Some of these problems can be overcome if the woman is informed antenatally about the benefits of breastfeeding and prepared mentally for exclusive breastfeeding. A randomized controlled trial conducted in a tertiary hospital in Singapore has revealed that antenatal breastfeeding education and postnatal lactation support, as single interventions based in hospital, both significantly improved rates of exclusive breastfeeding up to six months after delivery [1]. Lin et al have demonstrated the effectiveness of a prenatal education programme in Taiwan on maternal knowledge, attitude and satisfaction toward breastfeeding [2]. Breastfeeding support through an early, routine, preventive visit in the offices of trained primary care physicians has also been found to be effective in France [3]. Hence, antenatal counseling by trained health personnel has a role in promotion of breastfeeding.

In India, the health care provider during the antenatal visits is usually an obstetrician or an Auxiliary Nurse Midwife (ANM). In the rural health set up, ANM is the health functionary closest to the community. ANMs visit homes in their allotted villages and provide basic health services and health education including motivation of pregnant women to come to the sub-centre for initial check-ups and taking full course of iron and folic acid. Although opportunities exist during antenatal visits, counseling mothers regarding breastfeeding is often not done [4]. According to a study from Maharashtra, India, conducted in 1996–97, nearly half the pregnant women did not receive information regarding breastfeeding [5]. This deficiency is likely to affect the promotion and support of breastfeeding.

This study was undertaken to assess the awareness of breastfeeding information among primigravida mothers and the education on breastfeeding they had received during the antenatal visits in one tertiary hospital in southeastern India.

## Methods

This descriptive study was conducted at Mahatma Gandhi Medical College and Research Institute, Pondicherry from June to December 2005. On average, ten mothers deliver in the hospital daily and among them usually three are primigravid. Every third primigravid woman was recruited by a clinician (first author); only one woman was recruited each day because of time constraints. Among these 144 primigravida mothers, 108 who had a minimum of three antenatal visits ("booked") were included in the study. Initially a pilot study was conducted for 10 mothers using a questionnaire related to common health information regarding breastfeeding after which the questionnaire was modified. This pre-tested semi-structured questionnaire (see Additional file 1) on breastfeeding in the local language, Tamil, was administered to the mothers in the study group within 24 hours of delivery. Appropriate flash cards with pictures depicting good and poor attachment were also used while administering one question regarding the correct technique of breastfeeding. The main outcome variables were: i) Whether primigravid mothers have been informed about the benefits of breastfeeding during antenatal visits, ii) Whether they have acquired the basic health information about breastfeeding and iii) Whether breast examination was done during their antenatal visits. We considered a woman had received counseling if she responded that she had been informed of the benefits of breastfeeding at least once. Chi-square test was used to compare awareness of health information related to various aspects of breastfeeding among the mothers in both "counseled" group and "not counseled" group. The data were analyzed using statistical software package SPSS version 11.0. The Medical Ethics Committee of Mahatma Gandhi Medical College and Research Institute, Pondicherry approved the study. Informed consent was obtained from all participants.

## Results

The characteristics of mothers in both the "counseled" group and the "not counseled" group were similar. Their ages ranged from 19 to 23 years. All mothers were from rural areas and joint families and were either housewives or daily waged laborers. They had received formal school education from fourth to tenth grade. Of the 108 "booked" mothers, 23 (21%) had received antenatal counseling on breastfeeding while 85 (79%) had not received any such counseling. Only 4 (4%) booked mothers had undergone breast examination during antenatal visits. The awareness of health information related to various aspects of breastfeeding among the mothers in both "counseled" group and "not counseled" group is presented in Table 1. In the "counseled" group 87% were aware that breastfeeding should be initiated immediately after birth and 78% knew that exclusive breastfeeding should be continued for 6 months while in the "not counseled" group, only 19% and 22% were aware of the same, respectively. However, even in the "counseled" group awareness with regard to correct breastfeeding technique and concept of continuing breastfeeding during illness in the baby was no different from those in the "not counseled" group.

**Table 1:** Breastfeeding information known to "counseled" and "not counseled" mothers

Health Information	Mothers "counselled" (n = 23)		Mothers "not counselled" (n = 85)		p value
	Aware	%	Aware	%	
Initiate breastfeeding immediately after birth	20	87	16	19	< 0.01
Exclusive breastfeeding to be practiced for first 6 months	18	78	19	22	< 0.01
No prelacteal feeds to be given	23	100	38	45	< 0.01
Correct technique of breastfeeding*	1	4	0	0	0.48
No dietary restriction for lactating mother	10	43	6	7	< 0.01
Breastfeeding babies less than 6 months do not require extra water	8	35	3	4	< 0.01
Continue breastfeeding during common illnesses in baby	11	48	47	55	0.68

* Appropriate flash cards with pictures shown to mothers

## Discussion

The key to successful breastfeeding is likely to be Information, Education and Communication (IEC) strategies aimed at behavior change [6]. According to a study by Sable and Patton from Missouri, USA in 1989–91, only 37% of antenatal women reported that their health providers advised them to consider breastfeeding [7]. In our study, though 75% of the antenatal mothers were "booked", only 21% had received antenatal counseling on breastfeeding. It is evident that counseling on breastfeeding is not given due importance as part of antenatal visits. Though a trial by Alexander et al suggested that routine breast examination during antenatal care does not increase the chances of successful breastfeeding [8], detection of retractile nipples in the antenatal period followed by appropriate manoeuvres to make the nipples protractile may help in ensuring the success of breastfeeding in the postnatal period [4]. However, further research on this issue is required.

In this study, awareness related to breastfeeding among mothers in the "counseled" group was better than those in the "not counseled" group. We hypothesize that the women who received antenatal advice would be more likely to practice exclusive breastfeeding; however, only follow up studies can validate our hypothesis. Even in the "counseled" group awareness among mothers with regard to correct breastfeeding technique and concept of continuing breastfeeding during illness in the baby is not different from those in the "not counseled" group.

Where breastfeeding practices are suboptimal, simple one-encounter antenatal education and counseling may improve breastfeeding practice up to 3 months after delivery [9]. Provision of printed or audiovisual educational material is not enough. During antenatal visits, Health care providers should make every effort to have face-to-face encounter to give accurate information on breastfeeding and clarify misconceptions among expectant mothers. Health care providers also need education and training in breastfeeding support and management. Further research is needed to know why health care providers do not discuss the benefits of breastfeeding with women antenatally.

The Baby Friendly Hospital Initiative (BFHI), launched in 1991, is an initiative to ensure that all maternity services whether free standing or in a hospital, become centers of breastfeeding support [10]. A maternity facility can be accredited "Baby Friendly" when it does not accept free or low-cost breastmilk substitutes, feeding bottles or teats, and has implemented the Ten Steps to successful breastfeeding. Step Three is "Inform all pregnant women about the benefits and management of breastfeeding". According to Breastfeeding Promotion Network of India (BPNI), only 10% of hospitals and maternity facilities in India had BFHI status in 2005 [11]. This reflects the fact that more effort is needed to make all existing hospitals "Baby Friendly". Our hospital is also working towards accreditation. If appropriate measures are undertaken to strengthen training in breastfeeding counseling and the number of trained professional/peer counselors at all levels is increased, exclusive breastfeeding might become a social norm.

## Conclusion

Existing antenatal counseling on breastfeeding is inadequate in the population studied and needs to be strengthened. Informing all pregnant women about the benefits and management of breastfeeding should be a priority during antenatal visits.

## Abbreviations

BFHI: Baby Friendly Hospital Initiative; ANM: Auxiliary Nurse Midwife.

## Competing interests

The authors declare that they have no competing interests.

## Authors' contributions

GD was involved in designing the study and data collection and analysis. AB helped in data analysis and prepared manuscript. AA helped in data collection. SA supervised the study.

## Supplementary Material

Additional file 1 Antenatal counselling on breastfeeding – is it adequate? Questionnaire. Click here for file
