# Supplementary material for: Antenatal counseling on breastfeeding – is it adequate? A descriptive study from Pondicherry, India
Source: Int Breastfeed J. 2008 Mar 4;3:5. doi: 10.1186/1746-4358-3-5 (PMC2270808; doi:10.1186/1746-4358-3-5)
Supplement: Additional file 1 — Antenatal counselling on breastfeeding – is it adequate? Questionnaire. [file 1746-4358-3-5-S1.doc]

**Questionnaire used**

**Antenatal counseling on breastfeeding – Is it adequate?**

**Date: Village name: Sl.no:**

**1. Details about the mother:**

**Name Age: Religion:**

**Hospital Number:              In-patient Number:**

**Educational qualification: Occupation:**

**2. Details about the father:  Educational qualification:**

**Occupation:**

**3. Type of family               : Nuclear/Joint**

**4. Details about the antenatal visits:**

| **Trimester** | **No. of visits** | **Health Personnel who examined**  **(MBBS/Specialist/ANM)** | **Details of**  **Investigation / treatment** |
| --- | --- | --- | --- |
| **I** |  |  |  |
| **II** |  |  |  |
| **III** |  |  |  |
| **Total number of visits** |  |  |  |

**5. Was breast examination done during any visit?**

**If yes, any advice was given after the examination?**

**6. Were you informed about the benefits about the breast milk and exclusive breast feeding during any antenatal visit?**

**Mother’s response:**

| **Health Information** | **Yes** | **No** | **Don’t know** |
| --- | --- | --- | --- |
| Initiate Breastfeeding immediately after birth |  |  |  |
| Exclusive breastfeeding to be practiced for first 6 months |  |  |  |
| No prelacteal feeds to be given |  |  |  |
| No dietary restriction for lactating mother |  |  |  |
| Breastfeeding babies less than 6 months do not require extra water |  |  |  |
| Continue breastfeeding during common illnesses in baby |  |  |  |

Whether mother knows the correct technique of Breastfeeding? **(Yes / No)**

(Appropriate flash cards with pictures shown to mothers)
